# The Venereal Ulcer, or Chancroid

**Published:** 1877-05

**Authors:** Charles M. Allin

**Affiliations:** President, in the Chair


### Miscellaneous.

### New York Medical Journal Association---Stated Meeting, February 16, 1877.

Dr. Charles M. Allin, President, in the Chair.—The Venereal Ulcer, or Chancroid.

Dr. F. N. Otis read an interesting paper upon the above subject, of which the following is an abstract: The venereal ulcer, commonly called chancroid, was an acute contagious ulceration recognized as resulting from venereal contact. It was a purely local disease, and possessed characteristics which entitled it to be regarded as the highest type of acute ulcerative action. It commonly resulted from the inoculation of the purulent secretion of an ulcer having a similar character. Such purulent secretion, applied to sound integument or mucous membrane, was capable of effecting a solution of continuity, and of communicating to the sore at once its destructive and contagious properties. More commonly the chancroid was established upon an abrasion of the skin or mucous membrane in the act of coition. The secretion of the chancroid applied to an abrasion, congestion, inflammation, suppuration, and more or less rapid destruction of tissue followed in quick succession, and an ulcer was formed, sharply defined, with ragged edges, pultaceous floor, and secreting pus freely. The chancroid was seldom single, and occurring under circumstances of good general health, cleanliness and proper living, it was usually self-limited, and finally terminated in a scar such as was left after an ordinary burn. The secretion, from first to last, was purulent and inoculable, and the sore was capable of being reproduced as readily upon the person bearing it, as upon one free from the disease; but when thus artificially reproduced, it lost, to a certain extent, its contagious property in each successive inoculation, until at last the secretion was no longer inoculable. The tendency of the disease, therefore, under favorable conditions, was towards recovery. When, however, it was attended by unfavorable conditions, such as irregular life, filth, venereal and alcoholic excess, a depraved constiution, etc., the venereal ulcer assumed its most vicious type. It might then assume a high grade of inflammation and become *phagedenic*, or, in other and rarer instances, it might assume a sluggish type, known as the *serpiginous*, and persistently resist every mode of treatment for years. The extension of the chancroidal action might take place through the medium of the lymphatics and give rise to what was known as the chancroidal bubo. The contents of a bubonic abscess possessed all the peculiar properties of the pus from the original ulcer, and the open bubo took on the appearance of the typical chancroid, in its early stages, was easily controlled by proper remedial measures. The application of any caustic sufficient to destroy the affected tissue, sufficed to change the venereal into a simple ulcer, and recovery followed without farther treatment than such simple sores required.

In addition to the conditions mentioned as determining the severer forms of venereal ulcer, it was also to be recognized that the lesion present partook, in a great degree, of the activity, whether greater or less, which characterized the lesion from which it was derived. We therefore met with every grade, from the simple excoriation to the sharply defined and most active ulcer. Hence all cases did not require the energetic treatment necessary to arrest the typical venereal ulcer, or chancroid. The milder varieties required only mild measures, and simple antiseptic, sedative and astringent remedies might be sufficient to effect a rapid cure.

With regard to the

HISTORY OF CHANCROID

it was said to be conceded to be of ancient origin—even to antedate the advent of syphilis, which had been claimed in ancient Chinese and Asiatic records, and to have existed between two and three thousand years before the Christian era. Notwithstanding its early recognition, shortly after the introduction of syphilis in Europe, in 1494, it became confounded with that disease. Its purely local character was lost sight of, and it was subjected to constitutional treatment as a form of syphilis. From the time the confusion of the venereal ulcer and syphilis became complete, all the contagious venereal diseases, gonorrhœa, chancroid, or venereal ulcer, and syphilis, were practically regarded as identical—requiring the same constitutional treatment. For more than two hundred years forward it was supposed that constitutional syphilis followed gonorrhœa aud chancroid, and hence such patients were habitually mercurialized, but it finally became known that it was only the ulcer with indurated base and edge that was almost invariably followed by general syphilitic phenomena. John Hunter was the first publicly to recognize the value of the induration, characteristic of the venereal sore, which was followed by constitutional syphilis, thus making the first step towards restoring to the different venereal disorders their distinctive individuality. Hunter, however, was led into error, and taught that, while the local manifestations of venereal diseases were different their source of origin was identical, and that the variations depended upon some peculiar condition or idiosyncracy of the individual. In 1798 Benjamin Bell claimed a simple origin for gonorrhœa, and in 1836 Ricord, of Paris, by a series of experiments and observations, eliminated it from among the manifestations of syphilis. Ricord, however, accepted the view of Hunter, and regarded the hard and soft chancre as identical in origin, but made different by individual idiosyncracies. Bassereau, of Paris, one of Ricord's pupils, in 1852, demonstrated that in the soft local ulcer and the indurated infecting chancre, two distinct diseases existed. His observations were made by confrontation ; that is, by comparison of individuals, affected by venereal disease, with those from whom the disease had been acquired, and similar observations were made by Clerc, Diday, Rollet, and Fournier in 1856. In 1857 Fournier and Caby, directed by Ricord, proved that, in all cases of chancroid, the type of the ulcer remained unchanged in passing from one individual to another.

M. Clerc, although accepting and confirming the observations above alluded to, claimed to have demonstrated that the chancroid was the product of an inoculation of the syphilitic virus upon persons then or previously affected with syphilis, thus asserting a unity of origin for the two diseases. The same observer also claimed that, although as a rule the chancroid thus originated, transmitted only chancroid, yet on being inoculated upon a healthy person, it was capable of reverting to its original type and of communicating syphilis.

Rollet and others, on the contrary, held that the chancroid, the soft local sore, and the chancre, the initial lesion of syphilis, were separate and distinct diseases, and had their origin in separate and distinct influences. Thus the two schools, the unicists and the dualists, were initiated. Lee, of London, Bock, of Christiania, and others, succeeded in producing the typical chancroid upon persons syphilitic and non-syphilitic by inoculation of pus from an irritated syphilitic chancre. The degree of irritation required was that sufficient to produce free purulent secretion, and such sores were inoculable in successive generations upon persons quite free from syphilitic taint, and progressed in all respects like the ordinary venereal chancroid. Next it was found that when the superinduced irritation subsided, the secretion was no longer purulent; it was no longer auto-inoculable. It was concluded, therefore, that the property of inoculability or contagion depended upon a peculiar action resulting from the persistent irritation of an already diseased surface. The fact that such a sore could be established upon persons free from syphilitic antecedents, and not be followed by constitutional syphilis, demonstrated that it did not necessarily depend upon the syphilitic principle. Then came a series of experiments made by Pick, Koebner, Kaposi, and others, to ascertain the effect of inoculations of pus from simple lesions on persons free from syphilitic taint. The result had been formulated by Kaposi as follows: "Affections in non-syphilitic persons which are of slight virulence, and the secretions of which are not inoculable, can be made to produce an inoculable secretion by the application of an irritant." Reference was also made to the paper read by Dr. Bumstead before the Centennial Congress, in which was reported the case of Dr. Wigglesworth, of Boston, who in 1866, while in somewhat impaired health, inoculated himself with the pus from a simple acne pustule. Three generations of ulcers were established, and left as many distinct cicatrices. The experiment was under the personal observation of Prof. Zeissl, of Vienna. Reference was further made to the writings and experiments of Baumler, John Morgan, and Vidal. Personal observations had shown Dr. Otis that the muco-purulent secretion from a non-specific nasal catarrh would sometimes produce excoriation of sound cuticle; that contact with secretions from non-specific leucorrhœas would sometimes promptly cause pustular eruptions upon the preputial mucous membrane, more or less rapid in development, and progressing according to the degree of acridity of the fluid causing them—in some instances scarcely more than sero-purulent vesicles, in others so vicious that their development and progress did not differ appreciably from the typical chancroid. Reference was then made to several illustrative cases.

From all that had been adduced it seemed to Dr. Otis that it must be conceded the quality of pus was variable. and varied according to the circumstances under which it was produced, and the condition of the person on whom it might be inoculated. A low condition of the general system, from any cause, predisposed the tissues to take an ulcerative action and to elevate the attendant purulent secretion to a point of contagiousness. When, therefore, it came to be considered that the most frequent habitat of the venereal ulcer or chancroid was in localities where venereal excess and every kind of debauchery ran rampant; when to that was added the potent elements, syphilis, scrofula, filth, and irregular life, and also that chancroid was by far the most frequent, as compared with syphilis, among the debased and dissolute, the conclusion was regarded as inevitable that chancroid was of necessity a self-engendered disease, possessing no *specific* virus, but acquiring its power for destruction and contagion through various diseased conditions, or through the reckless stimulation and vitiation of benign natural processes. The distinction between chancroid and the initial lesion of syphilis, at the outset, was often impossible, and never possible unless the source of origin was known. The leading feature and characteristic of chancroid was a *necrosis*, that of the syphilitic lesion one of rapid *proliferation*. The latter was a *builder* of tissue, the former a *destroyer*. The only method of determining whether a given chancroid or other lesion was to be followed by constitutional syphilis (unless its source was known) was to wait at least one full month after the exposure, even though during that time the suspected lesion—possessing all the characteristics of the typical chancroid—had fully healed. The frequent association of chancroid with syphilis would never lead to mistaken identity if it was borne in mind that syphilis was always, in all its manifestations, the result of a process of *proliferation*, of exaggerated growth. The only product of syphilis, from the initial lesion to the growing tumor, was an excessive accumulation of tissue-building cells. Chancroid, on the other hand, from its inception to its cicatrization, was a process of *destruction* of tissue. It could be claimed, therefore, that syphilis and chancroid were always and only in relation to each other as life to death—each the highest type of its own peculiar action.

The paper being open to discussion—

Dr. F. R. Sturgis remarked with reference to the anti inoculability of non-specific pustules, such as acne, ecthyma, etc., that the experiments mentioned in the paper had been made upon persons who, at the time, were the subjects of debility, and that it was probably the debilitated condition which favored the development of the peculiar ulcers obtained ; whereas, with the inoculation of chanroidal ulcers, it made no difference, so far as debility was concerned. And it was only after repeated inoculations with the same virus had been made that there was a seeming immunity obtained ; but they could be reproduced with new virus in fresh soil as well as on the person bearing them.

The inoculation for acne, ecthyma, herpes, etc., had never been successful, unless the person upon whom the inoculation had been made was in a condition of debility. To inoculate healthy persons and produce an ulcer, the pustule must first be irritated, thereby introducing a new element into the experiment.

Dr. Sturgis had inoculated both himself and the persons bearing the lesion, from croton oil pustules, acne, scabies, and pemphigus, without the element of irritation being introduced, and on no occasion had the least effect been produced.

His experiments were performed upon those persons among whom they would be most likely to be successful, *i. e.*, hospital and dispensary cases, because they were surrounded by unfavorable conditions.

With regard to the statement made by Dr. Otis, that chancroid was probably not due to a special virus, but rather to unfavorable surrounding conditions, filth, excesses, irritation, etc., Dr. Sturgis believed that more evidence was required upon that point before it could be decided positively. Whether there was a real virus or not, we did not know what it was, and could, judging from its fruits, only say that it was some irritant which produced its peculiar effects.

Dr. Sturgis referred to a case mentioned by Dr. Otis in which the patient, not particularly strong, had been inoculated, with the probable production of a chancroid, and claimed that in such patients it was found that herpes would assume all the characteristics of a superficial chancroid, producing in the persons a lesion carrying out all the semblance of a chancroid, except that it was believed, if herpetic, to be incapable of inoculation either on the bearer or another ; and here he believed inoculation had not been practiced.

From the appearance of the two lesions one could not tell whether he had to deal with a superficial chancroid or a herpetic vesicle ; their semblance was such as to render it impossible to distinguish them. Dr. Sturgis believed that more light was required before it could be decided that acne pustules were inoculable in the same sense as chancroids.

Dr. Otis remarked that he wished to be understood as saying that sores on inoculation communicated like sores ; that a sore having a low grade of inoculability communicated one having a like low grade of inoculability, if any at all ; and that the chancroid in its typical state was a sore exposed to all the irritating influences which could be conceived of, and had thus been raised to its present degree of active virulence, but that it lost such activity by repeated inoculation.

It seemed to Dr. Otis that Dr. Sturgis had very happily supported him in the statement that sores which were engendered in weakly individuals produced on inoculation similar sores in personsalso debilitated, while no effect was produced when the secretion of the same sores was inoculated on healthy persons. From that fact it became evident that the contagious property was, to a certain extent, dependent upon the condition of the individual affected, and that debility was *one* of the known factors in producing the inoculable property in purulent secretions. It was further claimed that vinous and venereal excess, uncleanliness, etc., were among the other factors which went to make up the degree of virulence that characterized any given local contagious venereal lesion.

If we were compelled to wait until all the exact conditions obtaining in the production of typical chancroid were present in our experiments, the subject, of necessity, must remain open forever. . . . —*Med. Record.*

